# Effect of mixing and feed batch sequencing on the prevalence and distribution of African swine fever virus in swine feed

**DOI:** 10.1111/tbed.14177

**Published:** 2021-06-17

**Authors:** Catherine Grace Elijah, Jessie D. Trujillo, Cassandra K. Jones, Taeyong Kwon, Charles R. Stark, Konner R. Cool, Chad B. Paulk, Natasha N. Gaudreault, Jason C. Woodworth, Igor Morozov, Carmina Gallardo, Jordan T. Gebhardt, Jürgen A. Richt

**Affiliations:** ^1^ Department of Diagnostic Medicine/Pathobiology College of Veterinary Medicine Kansas State University Manhattan Kansas USA; ^2^ Center of Excellence for Emerging and Zoonotic Animal Disease Kansas State University Manhattan Kansas USA; ^3^ Department of Animal Sciences and Industry College of Agriculture Kansas State University Manhattan Kansas USA; ^4^ Department of Grain Science and Industry College of Agriculture Kansas State University Manhattan Kansas USA; ^5^ Animal Health Research Centre Instituto Nacional de Investigación y Technología Agraria y Alimentaria Madrid Spain

**Keywords:** African swine fever virus, bulk sampling, feed batch sequencing, feed safety

## Abstract

It is critical to have methods that can detect and mitigate the risk of African swine fever virus (ASFV) in potentially contaminated feed or ingredients bound for the United States. The purpose of this work was to evaluate feed batch sequencing as a mitigation technique for ASFV contamination in a feed mill, and to determine if a feed sampling method could identify ASFV following experimental inoculation. Batches of feed were manufactured in a BSL‐3Ag room at Kansas State University's Biosafety Research Institute in Manhattan, Kansas. First, the pilot feed manufacturing system mixed, conveyed, and discharged an ASFV‐free diet. Next, a diet was manufactured using the same equipment, but contained feed inoculated with ASFV for final concentration of 5.6 × 10^4^ TCID_50_/g. Then, four subsequent ASFV‐free batches of feed were manufactured. After discharging each batch into a collection container, 10 samples were collected in a double ‘X’ pattern. Samples were analysed using a qPCR assay for ASFV p72 gene then the cycle threshold (Ct) and Log_10_ genomic copy number (CN)/g of feed were determined. The qPCR Ct values (*p *< .0001) and the Log_10_ genomic CN/g (*p *< .0001) content of feed samples were impacted based on the batch of feed. Feed samples obtained after manufacturing the ASFV‐contaminated diet contained the greatest amounts of ASFV p72 DNA across all criteria (*p *< .05). Quantity of ASFV p72 DNA decreased sequentially as additional batches of feed were manufactured, but was still detectable after batch sequence 4. This subsampling method was able to identify ASFV genetic material in feed samples using p72 qPCR. In summary, sequencing batches of feed decreases concentration of ASFV contamination in feed, but does not eliminate it. Bulk ingredients can be accurately evaluated for ASFV contamination by collecting 10 subsamples using the sampling method described herein. Future research is needed to evaluate if different mitigation techniques can reduce ASFV feed contamination.

## 1 INTRODUCTION

The porcine epidemic diarrhoea virus (PEDV) outbreak of 2013–2014 was the first major disease outbreak to suggest a potential link between contaminated feed and pathogen transmission in pigs (Scott et al., 2016). This hypothesis was never unequivocally proven, but the concept of applying biosecurity practices to the United States swine industry feed manufacturing and delivery systems became heavily emphasized. Research has continued to demonstrate that the risk of feed‐based virus transmission extends beyond PEDV and could include viruses such as African swine fever virus (ASFV), foot‐and‐mouth disease virus (FMDV), or classical swine fever virus (CSFV) (Dee at al., 2018; Stoian et al., 2020). Improved biosecurity practices in the feed industry became particularly important in 2018, when a number of historically ASFV‐free countries in Southeast Asia began to report ASFV cases (Gaudreault et al., 2020). The United States maintains trade relationships with a number of countries that are now in ASFV‐endemic regions, leading to concerns that ASFV may enter the United States through the feed supply chain or other avenues. There is no active surveillance for ASFV in feed or ingredients imported from ASFV‐endemic regions, nor is there a validated protocol to sample or analyse for ASFV in a feed or ingredient matrix (USDA‐APHIS‐VS, 2019). It has been hypothesized that the same methods which demonstrated appropriate sensitivity and specificity for PEDV detection in feed may be applicable to ASFV, but this has not yet been tested. Furthermore, it has been suggested that mitigation measures common in PEDV, such as feed batch sequencing to reduce viral concentration, may be equally effective against ASFV. However, this has also never been evaluated. Therefore, the objectives of this study were to (1) determine if a common sampling strategy could consistently detect ASFV in feed, and (2) evaluate if feed batch sequencing could serve as a potential mitigation technique for ASFV contamination during feed manufacturing.

## 2 MATERIALS AND METHODS

### 2.1 General

The study was conducted at the Biosecurity Research Institute (BRI) at Kansas State University (KSU) in Manhattan, KS, with approval by the Kansas State University Institutional Biosafety Committee (project approval #1427.1). The feed manufacturing process was done within a biosafety level (BSL)‐3Ag animal room; the laboratory work was done within a BSL‐3+ laboratory space. Neither humans nor animals were used as research subjects in this experiment, so relevant approvals were not applicable.

### 2.2 Inoculation

To prepare the inoculum, 8.5 mL of pooled blood treated with ethylenediaminetetraacetic acid (EDTA) from ASFV‐infected pigs was mixed in RPMI media to prepare 530 mL of virus inoculum at a final concentration of 2.7 × 10^6^ TCID_50_/mL of ASFV genotype II virus (strain Armenia 2007).

### 2.3 Manufacture and sampling

Feed was manufactured as described by Schumacher et al. (2019). The feed manufacturing system was first primed with an ASFV‐free batch of feed, which was subsequently followed by a second batch of feed that was contaminated with ASFV. Four additional batches of ASFV‐free feed were subsequently mixed and discharged through the same equipment without any cleaning or disinfection occurring between batches. For this study, a corn and soybean‐meal‐based diet with a composition normally fed to gestating sows was manufactured at the Kansas State University O.H. Kruse Food Technology Innovation Center (Manhattan, KS) and transported to the BRI facility.

Treatments consisted of the following:

- Negative control (Batch 1)—Priming the feed mill: To initiate the trial, a 25 kg batch of ASFV‐free feed was mixed in a 50 kg capacity steel mixer with a 0.113 m^3^ electric paddle mixer (H.C. Davis Sons Manufacturing, model # SS‐L1; Bonner Springs, KS). The feed was mixed for 5 min, then discharged at a rate of approximately 4.5 kg/min into the conveyor (Universal Industries, Cedar Falls, IA) that carried 74 buckets (each 114 cm^3^) of feed. The feed was conveyed and discharged through a downspout into double‐lined bags.
- Positive control (Batch 2)—ASFV‐contaminated feed: Upon completion of priming the system with the initial batch of ASFV‐free feed, 530 mL of a genotype II ASFV (strain Armenia 2007) at a concentration of 2.7 × 10^6^ TCID_50_/mL was then mixed with 4.7 kg of feed in a 5 kg stainless steel mixer (Cabela's Inc., Sidney, NE) to make 5.23 kg of ASFV‐contaminated feed. This mixture was subsequently added to 20 kg of feed, resulting in a final ASFV concentration of 5.6 × 10^4^ TCID_50_/g, which was then mixed, conveyed, and discharged using the same equipment and procedures as previously described for the negative control.
- Sequences 1–4 (Batches 3, 4, 5, and 6)—Manufacture of subsequent batches of feed: Following the discharge of the ASFV‐contaminated batch of feed, the same process of mixing, conveying, and discharging 25 kg batches of feed was repeated four additional times using ASFV‐free feed.

After a batch of feed was discharged, 10 feed samples were collected just as previously described by Jones et al. (2020). Briefly, the 10 samples were taken from the feed that had been discharged in a biohazard tote through two ‘X’ patterns. To achieve this pattern, the biohazard tote was divided into two halves and in each half, two imaginary diagonal lines were drawn from corner to corner to make an ‘X’ pattern. Samples were taken from the corners of each half, along with a sample from the middle where the two imaginary diagonal lines crossed. The 10 samples were not mixed together, but were analysed in separate PCR reactions. This sampling technique resulted in a grand total of 60 feed samples for the entirety of the experiment.

### 2.4 Laboratory analysis

Feed samples were tested at a BSL‐3+ laboratory in the BRI. Briefly, 10 g of each feed sample was put in a tube, suspended with 35 mL of PBS, and the tube was capped and inverted, and then incubated overnight at 4°C. Approximately 10 mL of supernatant was recovered, aliquoted into 5 mL cryovials, and stored at −80°C until processed for qPCR. In preparation for magnetic bead‐based DNA extraction, 500 μL of PBS eluent was combined with 500 μL of Buffer AL (Qiagen, Germantown, MD, USA), briefly vortexed, and incubated at 70°C for 10 min in an oscillating heat block. DNA extraction was carried out using the GeneReach DNA/RNA extraction kit on a taco™ mini automatic nucleic acid extraction system (GeneReach, Boston, MA, USA). The extraction was performed according to the manufacturer's instructions, with modifications. Briefly, 200 μL of AL/sample lysate was transferred to column A of the taco™ deep‐well extraction plate which contained 500 μL of the GeneReach lysis buffer and 50 μL of magnetic beads, followed by an addition of 200 μL of molecular grade isopropanol (ThermoFisher Scientific, Waltham, MA, USA). The extraction consisted of two washes with 750 μL of wash buffer A, one wash with 750 μL of wash buffer B, and a final wash with 750 μL of 200 proof molecular grade ethanol (ThermoFisher Scientific). After a 5‐min drying time, DNA was eluted with 100 μL of elution buffer, and subsequently transferred into 1.5 mL of DNA/RNA‐free centrifuge tubes (VWR) for storage. Positive and negative extraction controls were included in sample processing, and consisted of a positive extraction control which was a partial sequence of the ASFV p72 gene cloned into plasmid Bluescript II and a negative extraction control, which was PCR‐grade water.

Real‐time quantitative PCR (qPCR) was carried out using primers and probes designed to detect the gene encoding for ASFV p72 and PerfeCTa FastMix II (Quanta Biosciences, Gaithersburg, MD, USA) on the CFX96 Touch Real‐Time PCR Detection System (Bio‐Rad, Hercules, CA, USA). The qPCR reactions were performed in duplicate, with each well containing 5 μL of template DNA, 0.2 μL (200 nM) of each primer (Integrated DNA Technology, Coralville, IA, USA), and 0.4 μL (200 nM) of FAM probe (Thermo Fisher Scientific) in a total reaction volume of 20 μL. Thermocycling conditions were 95°C for 5 min, followed by 45 cycles of 95°C for 10 s and 60°C for 1 min.

ASFV p72 genomic copy numbers (CN) were calculated using reference standard curve methodology using a reference standard curve composed from 10‐fold serial dilutions performed in triplicate of the quantitated ASFV p72 plasmid DNA control. The CN for samples was mathematically determined using the PCR‐determined cycle threshold (Ct) for ASFV p72 (two PCR well replicates) and the slope and intercept of the ASFV p72 DNA standard curve. Genomic CN/g feed for each sample was based upon the genomic CN/mL of solution recovered during sample processing, multiplied by the volume of PBS added during sample processing (35 mL), then divided by the amount of feed per suspension (10 g).

### 2.5 Statistical analysis

Statistical analysis for this study was performed using R programming language (Version 3.6.1 (2019‐07‐05), R Core Team, R Foundation for Statistical Computing, Vienna, Austria). The experimental unit for this study was the feed sample. Each feed sample had one extraction for the qPCR assay and each extraction was run in duplicate for qPCR analysis with the exception of samples from batch 2 in which each feed sample had two extractions for the qPCR assay; both extractions were run in duplicate for qPCR analysis as an initial assessment to evaluate the variability present within the extraction and amplification procedures.

Response values for the ASFV p72 gene were analysed using a linear mixed model fit using the lme function in the nlme package, using a normal distribution with the fixed effect as batch, with a random effect of sample to indicate the appropriate level of experimental replication given the duplicate qPCR analysis of feed samples. Results of Ct and genomic CN/g are reported as least squares means ± standard error of the mean. Samples not containing detectable ASFV DNA were assigned a value of 45 because this was the highest number of cycles the qPCR assay performed before concluding a sample did not have detectable ASFV DNA. Genomic CN/g data were Log_10_ transformed prior to data analysis to satisfy the assumption of normality. All statistical models were evaluated using visual assessment of studentized residuals and models accounting for heterogeneous residual variance were used when appropriate. A Tukey multiple comparison adjustment was incorporated when appropriate. Results were considered significant at *p* ≤ .05 and marginally significant between *p *> .05 and *p* ≤ .10.

## 3 RESULTS AND DISCUSSION

Outbreaks of PEDV in North America were the first events with a potential link between contaminated feed and transmission of disease to pigs (Scott et al., 2016). Since then, veterinarians, producers, and feed manufacturers have focused their efforts in preventing biological hazard transmission through the feed supply chain using both prevention and biosecurity strategies (Stewart et al., 2020; USDA‐APHIS, 2019). Potential solutions to ensure feed safety have routinely included different types of mitigation strategies, either utilizing these strategies alone, or in combination with each other to reduce potential PEDV contamination within the feed (Cochrane et al., 2015, 2017; Gebhardt et al., 2016 , Schumacher et al., 2019). It has become commonplace for swine producers to exclude feed or ingredients from countries that are endemic to viruses currently not present in the United States. However, this is sometimes difficult to implement because the United States relies on agricultural trade with countries that are endemic to ASFV. For example, the majority of the vitamins used in domestic swine diets are manufactured in ASFV‐endemic countries (Shurson et al., 2019). While their manufacture is typically in biosecure laboratories, and the ingredients themselves may pose low risk for foreign animal disease transmission, containers carrying these ingredients may become contaminated and thus become a potential source of ASFV entry into the United States. In theory, ingredients could be sampled for ASFV and screened for safety prior to entry into the country, but surveillance of this magnitude has not been implemented, partially due to the lack of validated bulk sampling or extraction methodologies (USDA‐APHIS‐VS, 2019). The Association of American Feed Control Officials (2014) and the Federal Drug Association (2021) recommend a similar method for sampling bulk containers to account for the unequal distribution of swine viruses, but this method still remains untested for many viruses. Jones et al. (2020) were successful in identifying PEDV in bulk containers utilizing this method, but this sample strategy remains unproven for other viruses like ASFV or FMDV. Because of this, one of the intents of this study was to determine if a common bulk sampling strategy could be used by US regulators to detect feed contaminant levels to consistently detect ASFV contamination. This study also wanted to evaluate a feed mitigation technique of feed batch sequencing, a technique that has been tested with PEDV, to see if this would be a potentially useful practice for ASFV‐contaminated feed.

After the ASFV‐positive batch of feed was manufactured, all feed samples had detectable ASFV p72 genetic material (Table 1). The number of samples with detectable ASFV p72 genetic material decreased with each subsequent batch. However, by sequence 4, feed samples still contained detectable ASFV p72 genetic material. In terms of the presence of ASFV DNA, the batch of feed impacted the Ct value (*p *< .0001) and the Log_10_ genomic CN/g (*p *< .0001; Table 2) of samples. Samples taken from the feed manufactured with direct contamination with ASFV contained the greatest amount of ASFV p72 genetic material across all response criteria (*p *< .05). Sequence 1 had slightly lower levels of ASFV DNA detected compared to the positive control batch (*p *< .05), and sequence 4 had a lower ASFV DNA quantity than both the positive control batch and sequence 1 (p< .05). The level of detectable ASFV DNA in sequences 2 and 3 were intermediate between sequences 1 and 4. In general, the quantity of detected ASFV p72 DNA decreased sequentially as additional batches of feed were manufactured. However, detection of ASFV p72 DNA was still possible after four sequences of ASFV‐free feed. This suggests that flushing a feed mill with ASFV‐free feed after an ASFV‐contaminated feed will reduce the amount of ASFV in the feed, but will not eliminate the virus entirely. Schumacher et al. (2019) found similar results in their study evaluating sequencing to reduce PEDV contamination. The current study's findings also suggest that the 'X' pattern sampling technique used was able to identify ASFV contamination within feed samples and supports the study by Jones et al. (2020) who had similar success with this sampling method for detecting various levels of PEDV contamination within feed containers. The probability of infection in the feed samples collected for this study could be estimated using the data on ASFV infectious dose and the probability of infection recently published by Niederwerder et al. (2019). Based on their ASFV exposure model, the amount of genomic CN/g found in this study's feed samples from sequences 1–4 has an infection probability ranging from 0.25 to 1.00. However, Niederwerder et al. (2019) used genotype II ASFV, but a different isolate (Georgia 2007/1) for their study, so infectivity based off their model is an extrapolation.

**Table 1:** Detection of African swine fever virus (ASFV) p72 DNA in feed samples

	Batch of feed^†^					
	Negative	Positive	Sequence 1	Sequence 2	Sequence 3	Sequence 4
Positive	0/10	10/10	10/10	9/10	9/10	7/10
Suspect	0/10	0/10	0/10	1/10	1/10	3/10
Non‐detected	10/10	0/10	0/10	0/10	0/10	0/10

^†^ Swine gestation feed was inoculated with African swine fever virus (ASFV) at 5.6 × 10^4^ TCID_50_/gram inoculated feed (positive) following an initial priming of the feed manufacturing equipment with ASFV‐free feed (negative). Four subsequent batches of feed were manufactured (sequence 1 to 4) and were initially free of ASFV. Ten feed samples were collected from each subsequent batch of feed and analysed using an ASFV p72‐specific qPCR assay with each sample analysed in duplicate. Samples were considered qPCR positive if 2 of 2 qPCR reactions had detectable ASFV DNA, suspect if 1 of 2 qPCR reactions had detectable ASFV DNA, and non‐detected if 0 of 2 qPCR reactions had detectable ASFV DNA.

**Table 2:** Concentration of detectable African swine fever virus (ASFV) p72 DNA in feed samples

	Batch of feed^†^					
Assay:	Negative	Positive	Sequence 1	Sequence 2	Sequence 3	Sequence 4
Cycle threshold^§^	45.0	33.0 ± 0.37^a^	37.5 ± 0.42^b^	39.5 ± 0.61^b,c^	39.3 ± 0.61^b,c^	40.1 ± 0.61^c^
Log_10_ genomic copies/g^¶^	0.0	4.7 ± 0.08^a^	3.6 ± 0.09^b^	3.1 ± 0.23^b,c^	3.1 ± 0.23^b,c^	2.8 ± 0.23^c^

^†^ Swine gestation feed was inoculated with African swine fever virus (ASFV) at 5.6 × 10^4^ TCID_50_/gram (positive), following an initial priming of the feed manufacturing equipment with ASFV‐free feed (negative). Four subsequent ASFV‐free batches of feed were manufactured (sequence 1 to 4). Ten feed samples were collected after each batch of feed and were analysed using an ASFV p72‐specific qPCR assay with each sample analysed in duplicate for each assay. Statistical analysis includes all treatment groups except for negative control where samples were collected prior to ASFV inoculation. Values for main effect of batch do not include negative batch of feed.

^§^ Cycle threshold values for qPCR reactions with no detectable ASFV p72 gene expression were assigned a value of 45 within the statistical analysis. Batch: *p* < .0001.

^¶^ Log_10_ transformed genomic copies for the ASFV p72 gene per g of feed from feed samples. Batch: *p *< .0001.

^a,b,c^Means within row lacking common superscript differ (*p* < .05) using Tukey multiple comparison adjustment.

A limitation of this experiment is the lack of infectivity data associated with the feed samples containing ASFV p72‐specific DNA. This research utilized ASFV, a BSL‐3 pathogen, and a US select agent; meaning, to get approval to use this virus is a rigorous progress, requiring special laboratories, and intensive training. Validating these feed samples for ASFV infectivity is important, and will be an area of our future research efforts; however, the focus of this study was to determine if feed sequencing was an effective mitigant strategy for ASFV‐contaminated feed and if feed sampling techniques could accurately identify ASFV genetic material. The data presented here provides significant value to the global feed and swine industry by establishing the presence of ASFV DNA in feed after first contaminating and then flushing a feed production system with subsequent batches of 'clean' feed, along with the ability to detect ASFV genetic material in the feed which can provide information for urgently needed surveillance programs.

In conclusion, sequencing with four batches of feed after contamination of a feed mill with ASFV can decrease overall ASFV contamination within feed samples, but not eliminate it entirely. In addition, collecting 10 evenly distributed samples using an 'X' pattern collection system allows for the detection of ASFV genetic material under the conditions of the current investigation. The findings of this study highlight the importance of excluding ingredients from ASFV‐endemic countries, but also highlights that proper sampling can be an effective tool to detect ASFV contamination. Additional research is necessary to evaluate the combination of mitigation techniques like chemically treating flush diets (similar to what is done with PEDV) on ASFV‐contaminated ingredients.

## CONFLICT OF INTEREST

The authors declare no conflicts of interest in the completion and reporting of results for this study.

## ETHICAL STATEMENT

The authors confirm that the ethical policies of the journal have been followed. No animal or human subjects were used in the study, and all study protocols were reviewed and approved by the Kansas State University Institutional Biosafety Committee.
